# Clinical Mastitis Incidence in Dairy Cows Housed on Recycled Manure Solids Bedding: A Canadian Cohort Study

**DOI:** 10.3389/fvets.2021.742868

**Published:** 2021-09-23

**Authors:** Annie Fréchette, Gilles Fecteau, Caroline Côté, Simon Dufour

**Affiliations:** ^1^Regroupement Fonds de recherche du Québec - Nature et Technologies (FRQ-NT) Op+lait, Saint-Hyacinthe, QC, Canada; ^2^Mastitis Network, Saint-Hyacinthe, QC, Canada; ^3^Department of Pathology and Microbiology, Faculty of Veterinary Medicine, Université de Montréal, Montreal, QC, Canada; ^4^Department of Clinical Sciences, Faculty of Veterinary Medicine, Université de Montréal, Montreal, QC, Canada; ^5^Institut de Recherche et de Développement en Agroenvironnement, Saint-Hyacinthe, QC, Canada

**Keywords:** recycled manure solids, dairy cows, bedding, housing, clinical mastitis

## Abstract

Bedding can affect mammary health of dairy cows. The objectives of this study were to evaluate clinical mastitis incidence in cows housed on recycled manure solids bedding and, more specifically, to determine which pathogens were involved. We followed 26 recycled manure solids farms and 60 straw-bedded farms as a comparative group during 1 year (2018–2019). For each episode of clinical mastitis, defined as a visual alteration of the milk, with or without local or systemic signs of infection, producers sampled aseptically the affected quarter, provided some details about the animal, and sent the sample to the research team at the Université de Montréal. We received and analyzed 1,144 milk samples. The samples were cultured according to the National Mastitis Council guidelines and the different colony phenotypes were subsequently identified with mass spectrometry. In 54.6% of CM cases, a single phenotype of bacteria was cultured (pure culture), while two different phenotypes were found in 16.8% of the samples (mixed culture), and no growth was observed in 14.4% of the samples. Samples with three or more phenotypes were considered contaminated and were not included in the pathogen-specific analyses (14.3% of the submitted samples). The most frequently identified bacterial species in pure and mixed culture in farms using recycled manure solids were *Streptococcus uberis* (16.0%), *Escherichia coli* (13.8%), *Klebsiella pneumoniae* (13.2%), *Streptococcus dysgalactiae* (6.2%), and *Staphylococcus aureus* (3.4%). In straw farms, the most frequent species were *S. aureus* (16.6%), *S. uberis* (11.0%), *E. coli* (9.1%), *S. dysgalactiae* (8.0%), and *K. pneumoniae* (1.1%). The incidence of clinical mastitis (all cases together) was not higher in recycled manure solids farms (14.0 cases/100 cow-year; 95% CI: 8.3–23.7) compared with straw-bedded farms (16.3 cases/100 cow-year; 95% CI: 9.0–29.6). However, *K. pneumoniae* clinical mastitis episodes were 7.0 (95% CI: 2.0–24.6) times more frequent in recycled manure solids farms than in straw farms. Adjusted least square means estimates were 1.6 *K. pneumoniae* clinical mastitis cases/100 cow-year (95% CI: 0.8–3.4) in recycled manure solids farms vs. 0.2 cases/100 cow-year (95% CI: 0.1–0.6) in straw-bedded farms. *Klebsiella pneumoniae* clinical mastitis is in general severe. Producers interested in this bedding alternative need to be aware of this risk.

## Introduction

Dairy producers are interested in alternative bedding products that may be less expensive to buy or produce, easily available in large quantities, and secure for animals and humans. There is a growing interest in Eastern Canada to use recycled manure solids (RMS) as bedding. This product is already used in many countries such as the United States, United Kingdom, and the Netherlands. However, there is no consensus on the best technique to produce RMS to ensure its safety. The method used to produce RMS and the different climates where it is used will influence its physicochemical and, possibly, its microbiological characteristics (1, 2). Recycled manure solids is an organic bedding with a high moisture content and, therefore, represents a favorable environment for bacterial growth. Indeed, previous studies demonstrated the high bacterial content of this product (3, 4) and its potential to sustain bacterial growth (5).

There are few studies on the association between RMS usage and animal health. It was demonstrated that *Cryptosporidium* parasites were found more frequently from the feces of cows in RMS farms compared with cows housed on straw bedding (6). In the same project, the presence of *Listeria monocytogenes* and *Salmonella* spp. was more frequently detected in RMS bedding samples, showing that the processing methods used to produce RMS were not efficient to eliminate these zoonotic pathogens^1^. There are also some concerns about the survival of *Mycobacterium avium* ssp. *paratuberculosis* throughout the RMS maturation process (7).

Associations between the use of RMS bedding and the risk of clinical mastitis (CM) were evaluated in two studies that yielded conflicting results. In an experimental study in one dairy facility of 309 primiparous Holstein cows, associations between type of bedding (RMS or sand) and CM incidence could not be highlighted (8). In a second study, 1,600 cows housed together in one farm were followed using an observational study design, and in this study, the use of RMS was associated with a greater risk of CM with an odds ratio of 2.1, compared with cows housed on sand (9).

There are anecdotal reports from veterinarians and producers about an increased incidence of CM and particularly CM caused by *Klebsiella* spp. on farms using RMS bedding. There are two main hypotheses explaining this potential increase in CM incidence. First, this bedding may contain an increased amount of *Klebsiella* spp. before usage compared with more conventional bedding. However, this hypothesis was refuted in two studies which observed that unused RMS, on average, contained less *Klebsiella* spp. than straw (4). Another possible explanation is that this CM may be due to the ability of *Klebsiella* spp. to multiply in this type of bedding during its use in the stalls. Indeed, the ability of RMS to support the growth of *Klebsiella pneumoniae* and *Enterococcus faecium* was investigated and shown to be superior to those of sand and wood products (5).

An increased incidence of *Klebsiella* spp. CM on farms using RMS was not yet confirmed in the scientific literature. Moreover, only one study have described CM incidence in a large number of farms using RMS bedding (10). Finally, pathogen-specific CM incidence was never reported on RMS farms. The objectives of this study were, therefore, to describe the total incidence of CM in dairy cows housed on recycled manure solids bedding and CM incidence by the main bacterial species and to compare these to herds using a more conventional type of bedding, straw. The results presented in this paper are part of a larger study on RMS farms, and results about milk quality, parasite survival, and bedding bacteriological analyses can be found elsewhere (6, 11).

## Materials and Methods

### Ethical Statement

This project was approved by the Animal Care and Use Committee of the Faculty of Veterinary Medicine (University de Montréal; protocol 17-Rech-1886). This paper was elaborated using the “STROBE-Vet statement” guidelines (12).

### Herd Recruitment

A list of farms using RMS was generated by contacting RMS equipment dealers and veterinarians and through social media. Straw farms were recruited with the help of Québec Dairy Herd Improvement Association (DHIA, Lactanet, Ste-Anne de Bellevue, QC, Canada). For both type of herds, to be eligible, farmers needed to be located within 250 km of the research facilities (St-Hyacinthe, QC), to have used the same bedding for >6 months prior to the farm visit, and for the straw farms, to be enrolled in a DHIA milk recording program. This latter condition was added for another part of the study on subclinical mastitis. We aimed at recruiting ~90 farms. This number was determined by an *a priori* power estimation. We estimated that, using 90 herds (20%: RMS bedding vs. 80%: straw bedding), milking an average of 50 cows, and a baseline probability of clinical mastitis of 20%, we would have >95% power to detect a difference of probability of mastitis corresponding to an odds ratio ≥1.4. For this calculation, we did not account for clustering of cows by herd. Thus, the real power is likely to be smaller than 95%.

All potential farms were contacted by telephone between July and December 2017 to verify their eligibility and willingness to participate. Basic demographic information such as the number of milking cows in the herd, type of bedding used, and for RMS farms, which equipment they used were also gathered from herds that were excluded, to study their similarities with participants and assess the presence of a selection bias.

### Sample Collection and Bacteriological Analyses

Farm visits were described elsewhere (6, 11). Briefly, farms were visited once and producers had to answer a standardized questionnaire about their bedding management. Methods used to produce bedding were recorded and bedding samples as well as bulk tank milk samples were collected. Herd size was recorded as the number of lactating cows. We also recorded housing type (free or tie stall), time since the last renovations of the stalls (in years), and bedding thickness defined as shallow bedding (<10 cm of depth) or deep bedding (≥10 cm of depth). These latter covariables were pre-identified using directed acyclic graph as putative confounders of the association between bedding type and CM incidence.

During 1 year following the initial visit, producers were asked to sample aseptically each quarter of cows experiencing a CM. Farmers had to provide information regarding the identification of the cow, its parity, the position of the quarter affected, and the severity of the CM. For the latter, farmers had to categorize CM events as score 1 (abnormal milk only), score 2 (abnormal milk and udder, without systemic signs), or score 3 (systemic signs of illness such as fever, depression, and anorexia) as described by Sears and McCarthy (13). Two consecutives cases of CM in the same quarter of a cow were considered distinct if they were ≥8 days apart (14). Samples were sent on ice to the Faculty of Veterinary Medicine Laboratory (St-Hyacinthe, QC, Canada). The bacteriological analyses were realized following the National Mastitis Council guidelines (15). Briefly, 0.01 ml of milk was plated on blood agar and incubated for 24–48 h at 35°C. The sample was then classified as negative (no growth), pure intramammary infection (IMI) (one single phenotype of CFU), mixed IMI (two types of CFU), or contaminated (≥3 types of CFU). An IMI was defined as the isolation of ≥100 CFU/ml of a given phenotype. Pure and mixed IMI bacterial isolates where then identified by mass spectrometry (MALDI-TOF) using the database of the manufacturer (BDAL-8468) and a custom database validated specifically for staphylococci identification (16). Isolates needed to be identified to the species level (vs. genus level solely) to be retained for pathogen-specific statistical analyses.

### Statistical Analyses

The number of CM episodes was compiled for each farm, as well as the number of severe (score 3) CM episodes. Finally, we compiled the CM episodes by specific pathogens. To account for the varying herd size and the exact time period of follow-up, the number of milking cows in each herd and the length of the follow-up period were also compiled.

Most CM studies have to deal with different levels of the compliance of producers for reporting CM cases and/or submitting samples. To investigate this potential bias, we used two different approaches to estimate the herd animal-time denominator used to adjust CM incidence. First, we used a common approach which is the exact number of milking cows and the exact period of follow-up (17) to compute the number of animal-year at risk of the herd.

Then, as a sensitivity analysis, we also estimated the follow-up period using the interval between the first and last sampling dates as the definition. Thus, with this alternative method, farms who did not send any samples or that sent only one sample during the 1 year study period were excluded (i.e., they would contribute 0 animal-year at risk). Moreover, farms that may have sent >one sample but then stopped sending samples at some point in time would be included, but with a shorter time at risk period (i.e., only the time between the first and last sent samples would be compiled). Then, we computed the number of cow-year at risk of the herd for each farm by multiplying the number of milking cows of the herd by the follow-up time. Using this alternative method allowed to exclude producers who sent <2 samples during the study period and weighted down producers who possibly stopped sending samples during the study.

Statistical analyses were performed using SAS 9.4 (SAS Institute Inc., Cary, NC, USA). Descriptive statistics were used to explore relations between predictors. To compare the incidence of CM in RMS and straw farms, we used a binomial negative model with the number of CM cases on a given farm as the outcome (firstly total number of CM cases, then severe CM cases only, then CM cases by bacterial species), type of bedding used (RMS or straw) as the main predictor, and the natural logarithm of the number of cow-year at risk as an offset term. In this model, we also included a number of putative confounding variables as predictors: housing type (free or tie stall), time since the last renovations of the stalls (in years), bedding thickness (deep or shallow bedding), and herd size (number of milking cows). With such a model, we could thus compute the CM incidence ratio (IR) between RMS- and straw-bedded farms, after adjusting for these confounders, simply by exponentiating the bedding coefficient. Moreover, the mean estimated CM incidence (in cases/100 cow-year) for a given type of herd could be computed simply by adding the intercept and the coefficients corresponding to that farm description, then exponentiating the results and, finally, multiplying the results by 100 cows (to obtain an incidence per 100 cow-year). Finally, all models were ran twice, initially using the complete follow-up period to compute the animal-time at risk of the herd and, then, using the animal-time at risk computed using the alternative method.

The assumption of linearity of the relation between quantitative predictors (time since the last renovation of the stalls and herd size) and the outcome (logarithmic transformation of the incidence ratio) was verified with the addition of polynomial terms (square and cubic terms) after centering the predictor. If the polynomial terms were significant (*p* < 0.05), the polynomial presentation of the variable was retained in the final model. If overdispersion was observed in the data (Pearson chi-square > 1.2), robust variance was used. Significance level was fixed at *p* ≤0.05. Data and the SAS code used to construct the models are publicly available at https://doi.org/10.5683/SP2/KIEMHY.

## Results

### Herds Description

We obtained a list of 49 RMS and 139 straw farms and recruited 27 and 61 RMS and straw farms, respectively. Reasons for exclusion of RMS farms were as follows: four had recently changed their bedding type to non-RMS bedding, 11 were outside the defined geographic location, one did not use RMS under the milking cows, and six could not be reached despite several attempts. From the 139 straw farms interested in the project, 61 were selected on their ability to provide computerized health records.

We visited the farms between January 15th and July 10th, 2018. The farms recruited have been described in Lasprilla-Mantilla et al. (6) and Gagnon et al. (11). Briefly, recruited RMS farms had 55–900 lactating cows (median 111) and straw farms had 43–229 lactating cows (median 65). An automatic milking system was in used in 37% of RMS farms and in 3% of straw farms. Furthermore, 59% of RMS farms and 98% of straw farms participated regularly in a DHI program. Even though participating in a DHI program was an inclusion criterion in straw herds, one farm did not record any data (i.e., dropped out of DHI) during the follow-up period. In the 27 RMS farms, 26 used a separation process as first step for producing RMS bedding. One RMS farm used an anaerobic digester as the first step and a separation process as the second and last step. From the 26 farms who did a separation first, one used the solid fraction immediately after separation, two used a rotative drum to turn into compost the solid fraction, 10 allowed the solid fraction to mature in a heap, and 13 allowed the solid fraction to mature in an enclosed container. During the monitoring year, two farms (one RMS and one straw) burned and five others (one RMS and four straw) dropped out of the project. Two of these farms (one RMS and one straw) dropped off very early and without sending any milk samples. They were, therefore, excluded from the analyses. For the three other farms, data collected until they left the project were used in the analyses. When we used the alternative follow-up period (from the first to the last sampling dates) to compute the animal-time at risk, 28% of RMS farms and 35% of straw-bedded farms had a follow-up time of 6 months or less. The characteristics of the general herds are reported in Table 1.

**Table 1:** Description of the 26 recycled manure solids farms and 60 straw farms.

	**RMS bedding**	**Straw bedding**
	**Median (range)**	**Median (range)**
Follow-up period in years	1.0 (0.4–1.0)	1.0 (0.2–1.0)
Number of milking cows	111 (55–900)	65 (43–229)
Number of years since the last renovations of the stalls	3.0 (0.1–23.0)	10.0 (0.0–70.0)
Proportion of freestall	70.4^a^	3.3^a^
Proportion of deep bedding (≥10 cm)	38.5^a^	0.0^a^

a *Proportion (in %)*.

### Milk Samples

**Figure 1:**
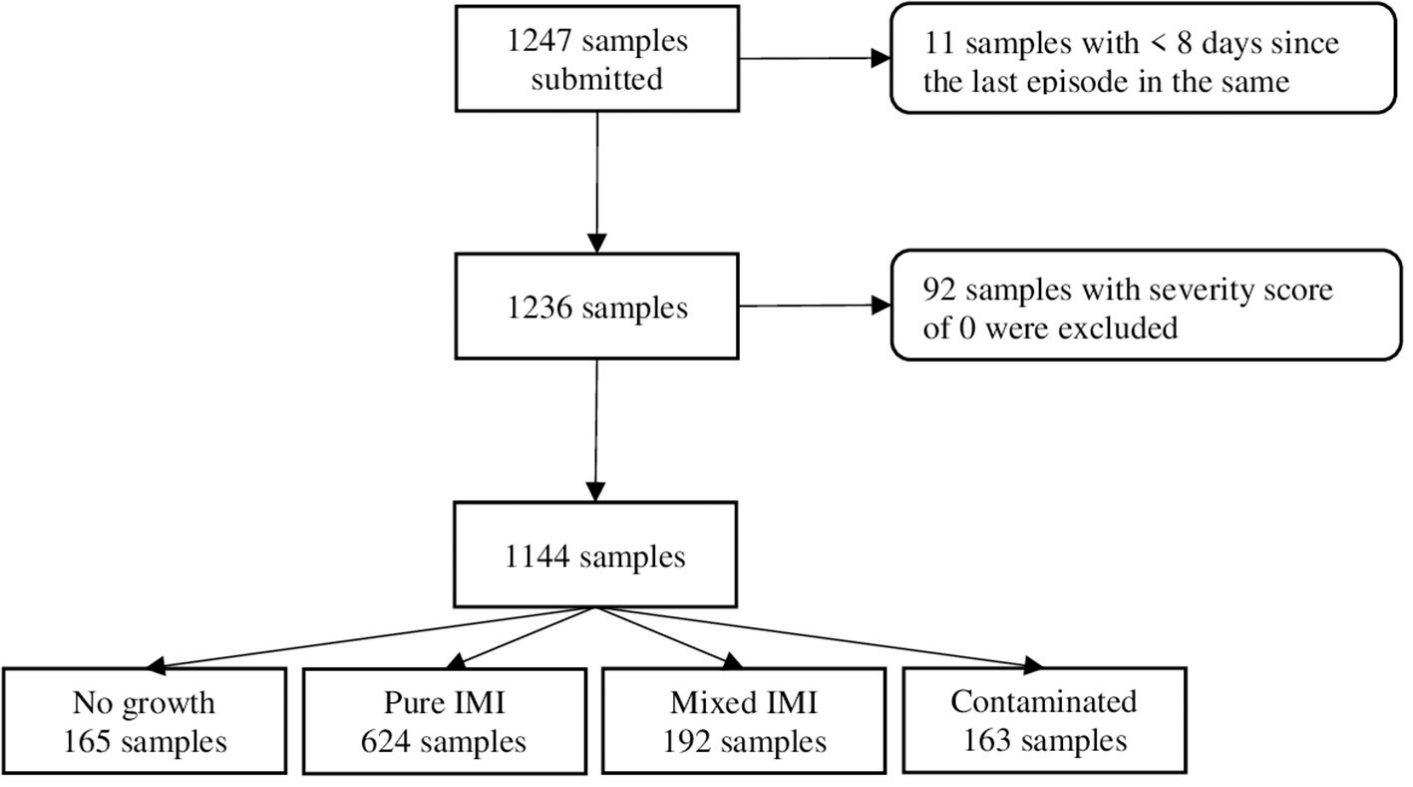
Flow chart representing clinical mastitis samples submitted and retained in a study comparing clinical mastitis incidence in 26 farms using recycled manure solids bedding and 60 farms using straw bedding.

We received 1,247 samples during the study period (Figure 1). We excluded 11 samples because they were collected on the same mammary gland quarter <8 days since the last CM episode. From the 1,236 remaining samples, there were no information about CM severity in 69 (5.6%) of the samples, 92 (7.4%) were submitted as score 0 (no clinical mastitis), 492 (39.8%) as score 1, 426 (34.5%) as score 2, and 157 (12.7%) as score 3. We excluded the 92 samples for which the producers explicitly reported a severity score of 0. Samples with no reported severity were, however, retained. We observed a pure IMI in 624 (54.5%) of the 1,144 remaining samples, a mixed IMI in 192 (16.8%), and no growth in 165 (14.4%). Of the collected samples, 163 (14.2%) were considered contaminated. The proportion of contaminated samples was not associated with bedding type (chi-square test; *p* = 0.89).

The most frequent recovered pathogens (in pure or mixed IMI) by bedding type and severity are reported in Table 2. Briefly, in RMS farms, the most frequent pathogens were *Streptococcus uberis* (16.0%), *Escherichia coli* (13.8%), *K. pneumoniae* (13.2%), *Streptococcus dysgalactiae* (6.2%), and *Staphylococcus aureus* (3.4%). In straw-bedded farms, *S. aureus* (16.6%), *S. uberis* (11.0%), *E. coli* (9.1%), *S. dysgalactiae* (8.0%), and *K. pneumoniae* (1.1%) were the most frequent pathogens. Clinical mastitis episodes due to coliforms (*K. pneumoniae* and *E. coli*) were more often severe. Clinical mastitis episodes due to *S. uberis, S. dysgalactiae*, or *S. aureus* were, in general, mainly mild or moderate (Table 2).

**Table 2:** Percentage (number) of clinical mastitis cases by bedding type, bacterial species, and severity among 1,144 clinical mastitis cases obtained on 26 farms using recycled manure solid (RMS) bedding and 60 farms using straw bedding.

**Category**		**RMS bedding**					**Straw bedding**			
	**Total** ^**a**^	**Severity** ^**b**^				**Total** ^**a**^	**Severity** ^**b**^			
		**Unknown**	**Mild**	**Moderate**	**Severe**		**Unknown**	**Mild**	**Moderate**	**Severe**
All cases	100 (356)	13.2 (47)	28.7 (102)	34.3 (122)	23.9 (85)	100 (788)	2.8 (22)	49.5 (390)	38.6 (304)	9.1 (72)
*Streptococcus uberis*	16.0 (57)	7.0 (4)	28.0 (16)	59.6 (34)	5.3 (3)	11.0 (87)	1.1 (1)	37.9 (33)	57.5 (50)	3.4 (3)
*Escherichia coli*	13.8 (49)	8.2 (4)	10.2 (5)	32.7 (16)	49 (24)	9.1 (72)	1.4 (1)	12.5 (9)	37.5 (27)	48.6 (35)
*Klebsiella pneumoniae*	13.2 (47)	6.4 (3)	8.5 (4)	31.9 (15)	53.2 (25)	1.1 (9)	0 (0)	33.0 (3)	33.0 (3)	33.0 (3)
*Streptococcus dysgalactiae*	6.2 (22)	18.2 (4)	40.9 (9)	22.7 (5)	18.2 (4)	8.0 (63)	0.0 (0)	34.9 (22)	55.6 (35)	9.5 (6)
*Staphylococcus aureus*	3.4 (12)	8.3 (1)	33.3 (4)	25 (3)	33.3 (4)	16.6 (131)	3.8 (5)	55.7 (73)	37.4 (49)	3.1 (4)
Other pathogen	19.4 (69)	23.2 (16)	34.8 (24)	26.1 (18)	15.9 (11)	28.6 (225)	2.7 (6)	57.8 (130)	35.1 (79)	4.4 (10)
No growth	16.9 (60)	6.7 (4)	48.3 (29)	30 (18)	15.0 (9)	13.3 (105)	1.9 (2)	61.0 (64)	28.6 (30)	8.6 (9)

a *Proportion of clinical mastitis cases in % (absolute number of cases) within a type of farm where a given bacterial species was found. Note that the sum within a column may add to more than 100% since some samples may yield more than one bacterial species*.

b *Distribution of severity of clinical mastitis cases in % (absolute number of cases) within a type of farm and for a given bacterial species. Severity was scored as described before (13) as follows: unknown, mild (1; abnormal milk only), moderate (2; abnormal quarter), or severe (3; abnormal cow)*.

### Effect of Bedding on CM Incidence

**Figure 2:**
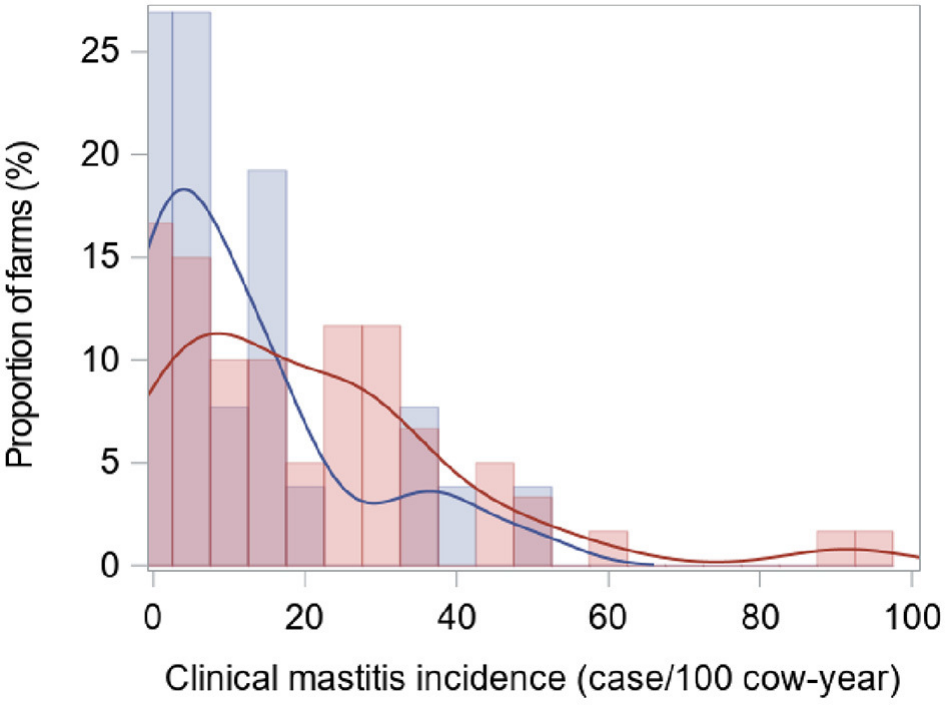
Clinical mastitis incidence distribution estimated using a period at risk extending from start to end of the study in a study comparing 26 farms using recycled manure solids bedding (blue) and 60 farms using straw bedding (red).

Clinical mastitis (all cases) incidence distribution estimated using a period at risk extending from start to end of the study is illustrated in Figure 2. Unconditional least square means (LSM) estimates (i.e., not adjusted for putative confounding variables) are presented as Supplementary Table 1. After adjusting for potential confounders, there was no statistical difference in the general CM incidence between the two farm types. A LSM estimate of 14.0 cases/100 cow-year (95% CI: 8.3, 23.7) was obtained for RMS farms, whereas a LSM of 16.3 cases/100 cow-year (95% CI: 9.0, 29.6) was obtained for straw farms (Table 3). Moreover, there was no difference in the incidence of severe CM episodes (severity score 3) between the two types of farm. A LSM of 2.1 severe cases/100 cow-year (95% CI: 1.1, 4.1) was obtained for RMS farms, and straw farms had a LSM of 1.6 severe cases/100 cow-year (95% CI: 0.8, 3.4).

**Table 3:** Least square means clinical mastitis incidence estimates (in cases/100 cow-year) and incidence ratio (IR) between 26 RMS farms and 60 straw-bedded farms and computed using a binomial negative model.

**Clinical mastitis category**	**Estimated incidence in cases/100 cow-year (95% CI)**		**IR (95% CI)**
	**RMS farms**	**Straw farms**	
All clinical mastitis	14.0 (8.3–23.7)	16.3 (9.0, 29.6)	0.9 (0.4–1.9)
Severe clinical mastitis	4.0 (2.5–6.3)	2.7 (1.6–4.6)	1.5 (0.7–3.1)
By bacterial species involved			
*Klebsiella pneumoniae*	1.6 (0.8–3.4)^a^	0.2 (0.1–0.6)^b^	7.0 (2.0–24.6)
*Streptococcus dysgalactiae*	0.6 (0.2–1.2)	0.8 (0.3–1.8)	0.7 (0.3–1.9)
*Escherichia coli*	0.5 (0.2–1.2)	0.7 (0.3–1.5)	0.8 (0.3–2.0)
*Streptococcus uberis*	0.4 (0.1–1.5)	0.8 (0.3–2.5)	0.5 (0.1–2.3)
*Staphylococcus aureus*	0.4 (0.1–1.2)	0.9 (0.3–3.0)	0.4 (0.1–1.4)

*A period at risk extending from start to end of the study was used to compute the animal-time denominator. Models were adjusted for confounding by housing type, time since the last renovation of the stalls, bedding thickness, and herd size. Means within a row with different superscripts are statistically different*.

The estimated incidence of *K. pneumoniae* CM, however, was higher in RMS farms with a LSM of 1.6 cases/100 cow-year (95% CI: 0.8, 3.4) compared with 0.2 cases/100 cow-year (95% CI: 0.1, 0.6) for straw farms. For this comparison, a 7.0 times (95% CI: 2.0, 24.6) higher incidence was observed in RMS farms. There was no significant difference between the two types of farms regarding the incidence of CM due to *S. uberis, E. coli, S. dysgalactiae*, or *S. aureus*.

### Sensitivity Analysis

When using the alternative approach for computing period at risk, we ended up excluding 8 RMS farms and 11 straw-bedded farms that sent <2 samples during the study period (Table 4). Using this alternative approach, the general estimated CM incidence was different between the two groups (Supplementary Table 2) with a LSM estimate of 26.5 cases/100 cow-year (95% CI: 19.2, 36.6) for RMS farms and a LSM of 46.2 cases/100 cow-year (95% CI: 30.2, 70.8) for straw-bedded farms. Furthermore, the total incidence of severe CM was not different between the two farms groups with a LSM estimate of 10.2 cases/100 cow-year (95% CI: 6.9, 15.0) in RMS farms and a LSM of 13.6 cases/100 cow-year (95% CI: 7.1, 26.1) in straw-bedded farms.

**Table 4:** Variation of the number of cows-year at risk in the two groups (26 RMS farms and 60 straw farms) when using different follow-up periods.

		**RMS bedding**	**Straw bedding**
		**Median (range)**	**Median (range)**
Cow-year at risk	Method 1	109 (55–900)	64 (10–229)
	Method 2	63 (0–230)	45 (0–109)

*Method 1 corresponds to a follow-up period of 1 year, and method 2 is a follow-up period calculated as the interval between the first and last sampling dates*.

As in our first approach, the estimated incidence of CM due to *K. pneumoniae* was significantly higher in RMS farms with a LSM of 3.4 cases/100 cow-year (95% CI: 1.6, 7.1) compared with 0.6 cases/100 cow-year (95% CI: 0.2, 1.6) in straw farms. This was equivalent to a 5.9 times (95% CI: 1.6, 21.2) higher incidence in RMS farms. There was still no difference between the two groups concerning the incidence of CM due to *S. uberis, E. coli, S. dysgalactiae*, or *S. aureus*.

## Discussion

To our knowledge, this is the first study to report pathogen-specific CM incidence on RMS farms. In our study, the proportion of contaminated samples was similar to a previously published work (18) and was not associated with the bedding type. Using the approach most often used in mastitis research to compute total CM incidence (i.e., considering the complete time period of follow-up), we did not observe a statistically significant association between the use of RMS as bedding and the incidence of CM. This finding is in agreement with those of an experimental study realized at the University of Wisconsin-Madison (8). In both studies, quarter milk was sampled and analyzed for each CM event, rather than just relying on the farm records for analysis of incidence. In the Rowbotham and Ruegg (8) study, environmental *Streptococcus, E. coli*, and *Klebsiella* spp. were identified in 50% of CM culture-positive samples. In that latter study, however, pathogen-specific CM incidence was not reported.

In our study, we were able to investigate the most common CM pathogens. Prior to conducting this study, veterinarians and producers in our province anecdotally reported *K. pneumoniae* CM outbreaks in RMS farms. This hypothetical higher incidence of *K. pneumoniae* CM in RMS farms was confirmed in the current study. One hypothesis to explain these mastitis episodes is that, right from the start, RMS bedding may contain a higher concentration of *K. pneumoniae* than straw. In a parallel study conducted on the same herds during the same period, we observed that unused RMS contained lower concentrations of *Klebsiella* spp. than unused straw. However, at the end of the usage cycle (prior to removal from the stall), the concentrations of *Klebsiella* spp. were similar between the two bedding types. Another hypothesis is that the growth rate of *Klebsiella* spp. would be higher in RMS bedding than in other bedding types. Thus, despite lower bacterial concentrations to start with, the rapid growth of *Klebsiella* spp. in this bedding type after contamination with feces would quickly lead to increased concentrations of *Klebsiella* spp. A previous study demonstrated the high potential of RMS for supporting the growth of *Klebsiella* spp. (5). Still, Beauchemin et al. also reported similar concentrations of *Klebsiella* spp. in samples using RMS and straw bedding. Thus, RMS did not seem to lead to a riskier environment (based solely on bacterial concentration) for *Klebsiella* spp. CM, even at the end of the usage cycle. Some other properties of this bedding, for instance its ability to stick to the teats, may better explain the higher *K. pneumoniae* CM incidence. This latter hypothesis, however, was not investigated in the current study.

Clinical mastitis episodes due to *K. pneumoniae* are usually severe (19). In our study, CM episodes due to *K. pneumoniae* were moderate or severe in 66% of cases in straw-bedded farms and in 85% of cases in RMS farms. In one study, they estimated a loss of 700 kg of milk in a multiparous cow experiencing clinical mastitis due to *Klebsiella* spp. at 30 days in milk (20). In comparison, a CM due to *E. coli* was causing a loss of 354 kilos. Moreover, cows experiencing *Klebsiella* spp. CM had a 22.3 times greater risk of culling than healthy cows (21).

Nevertheless, in our study, there was no difference in the total incidence of severe CM episodes between the two types of bedding. Since *K. pneumoniae* is just one of the multiple bacterial species that can cause severe CM, this result is not surprising. For instance, *E. coli* is another pathogen that was responsible for severe mastitis and *E. coli* CM incidence was similar between the two types of beddings.

When we used the alternative follow-up period (from the first to the last sampling dates) to compute the animal-time at risk, there were significantly more cases of CM in straw farms. Since herd size varied as function of bedding type, the impact of this more restrictive follow-up period affected differentially the incidence denominator for straw- and RMS-bedded herds. Consequently, since our results on the total CM incidence are affected by the method used to compute them, we can hardly conclude on whether the general CM incidence varied between the two groups of farms. The most commonly used approach in CM research (i.e., considering herds with few CM cases reported as herds with a low CM incidence) would conclude on similar CM incidence between bedding types. The more conservative approach (where herds reporting few CM cases or reporting for a limited period of time would be considered non-compliant and excluded) would conclude to a larger general CM incidence in straw-bedded farms. Nevertheless, regardless of the method used, the general CM incidence was never higher in RMS herds. Moreover, our results on species-specific CM incidence appeared to be robust and consistent between both computation methods.

A strength of our study was the number of participating farms and the number of cows recruited. To our knowledge, this is the largest number of herds and cows assembled to study the effect of RMS bedding on CM incidence. This is also the first time, to our knowledge, that pathogens responsible of CM were identified. We can now confirm that there are some differences in the pathogen patterns causing CM according to the bedding type. However, since this is an observational study, our study presents some limitations.

First, the sampling strategy was not random and some regions were overrepresented due to our proximity criteria. However, to our knowledge, most of the farms using RMS bedding in these regions and during this period were recruited. The bias may be more important regarding recruitment of farms using straw bedding, since we selected only herds enrolled in DHIA for that group. This criterion was not used for RMS farms. Producers enrolled in DHIA may be more concerned about udder health of their cows than the general population of dairy farmers, possibly generating a bias when measuring the association between bedding used and CM incidence. Nevertheless, a good proportion of RMS farms were also enrolled in DHIA, thus limiting the magnitude of this potential bias.

Second, the exposition to each type of bedding was not randomly assigned and many confounding factors were possibly operating within these farms. We were able to include in our models some potentially important confounding factors. Thus, our incidence estimates were adjusted for some of the other differences that we observed between RMS- and straw-bedded farms. Nevertheless, some residual confounding is likely to be present. Our results would have to be confirmed using an experimental study design where cows from one or many farms would be randomly assigned to different bedding types while monitoring pathogen-specific CM incidence.

Finally, a well-known challenge in studies on CM is the relatively low compliance of farmers for recording CM episodes, which may represent an information bias. We hypothesize that this lack of reporting was similar in the two groups of farms. In our study, to improve the reporting of CM episodes, we covered the costs for all the milk analyses conducted during the year of follow-up, provided timely results (i.e., <2 days) to the herd veterinarian, and called all participants every 4 months to keep them engaged and motivated. Moreover, using the alternative method for computing time of follow-up allowed for the exclusion of some herds that were possibly low-compliance herds.

In the future, experimental studies could help in confirming the results observed in this observational study. For instance, a randomized controlled trial or a crossover study design conducted in one or a few large herds and over a sufficiently long period of time (since *Klebsiella* spp. CM is an uncommon health event) would be of great value to confirm these initial findings.

## Conclusion

The general incidence of CM and of severe CM was not higher in RMS- compared with straw-bedded herds. However, the distribution of bacterial species causing the CM cases was different. The incidence ratio of CM due to *K. pneumoniae* was seven times greater in RMS farms than in straw farms. These mastitis cases are usually very severe. Producers interested to adopt this type of bedding must be aware of this risk.

## Data Availability Statement

The datasets presented in this study can be found in online repositories. The names of the repository/repositories and accession number(s) can be found at: https://doi.org/10.5683/SP2/KIEMHY.

## Ethics Statement

The animal study was reviewed and approved by Animal Care and Use Committee of the Faculty of Veterinary Medicine, University de Montréal. Written informed consent was obtained from the owners for the participation of their animals in this study.

## Author Contributions

All authors listed have made a substantial, direct and intellectual contribution to the work, and approved it for publication.

## Funding

This project was funded by grants from Novalait, the Consortium de recherche et innovations en bioprocédés industriels au Québec, the Fonds Québécois de la recherche sur la nature et les technologies (2017-LG-201835), and the Natural sciences and engineering research council of Canada (CRDPJ 499421 - 2016). The first author (AF) received funding and support from the Natural Sciences and Engineering Research Council of Canada Collaborative Research and Training Experience program in milk quality, from the Canadian Dairy Commission, from Agria, and from Op+lait.

## Conflict of Interest

The authors declare that the research was conducted in the absence of any commercial or financial relationships that could be construed as a potential conflict of interest.

## Publisher's Note

All claims expressed in this article are solely those of the authors and do not necessarily represent those of their affiliated organizations, or those of the publisher, the editors and the reviewers. Any product that may be evaluated in this article, or claim that may be made by its manufacturer, is not guaranteed or endorsed by the publisher.

## Footnotes

^1^Beauchemin J, Fréchette A, Thériault W, Dufour S, Fravalo P, Thibodeau A. Comparison of microbiota of recycled manure solids and straw bedding used in dairy farms in Eastern Canada. *J Dairy Sci*. (2021).
