# Supplementary material for: Clinical Mastitis Incidence in Dairy Cows Housed on Recycled Manure Solids Bedding: A Canadian Cohort Study
Source: Front Vet Sci. 2021 Sep 23;8:742868. doi: 10.3389/fvets.2021.742868 (PMC8495071; doi:10.3389/fvets.2021.742868)
Supplement: Supplementary file 1 [file Table_1.DOCX]

**Table S1.** Unconditional least square means clinical mastitis incidence estimates (in cases/100 cow-year) and incidence ratio (IR) between 26 RMS farms and 60 straw-bedded farms and computed using a binomial negative model. A period at risk extending from start to end of study was used to compute the animal-time denominator. Means within a row with different superscripts are statistically different.

| Clinical mastitis category | Estimated incidence  in cases/100 cow-year (95%CI) | | IR (95%CI) |
| --- | --- | --- | --- |
|  | RMS farms | Straw farms |  |
| All clinical mastitis | 11.8 (7.5, 18.6) | 20.7 (15.3, 27.9) | 0.6 (0.3-1.0) |
| Severe clinical mastitis | 4.6 (3.2, 6.7) | 2.8 (2.1, 3.8) | 1.6 (1.0, 2.6) |
| By bacterial species involved |  |  |  |
| *Klebsiella pneumoniae* | 1.8 (0.9, 3.2)^a^ | 0.2 (0.1, 0.4)^b^ | 8.4 (3.2, 22.3) |
| *Streptococcus dysgalactiae* | 0.7 (0.4, 1.2^)a^ | 1.6 (1.1, 2.3)^b^ | 0.4 (0.2, 0.9) |
| *Escherichia coli* | 1.2 (0.7, 2.1) | 1.8 (1.2, 2.5) | 0.7 (0.4, 1.3) |
| *Streptococcus uberis* | 1.3 (0.7, 2.6) | 2.0 (1.2, 3.1) | 0.7 (0.3, 1.5) |
| *Staphylococcus aureus* | 0.4 (0.2, 0.8)^a^ | 3.5 (2.5, 5.0)^b^ | 0.1 (0.0, 0.2) |
