# Supplementary material for: Clinical Mastitis Incidence in Dairy Cows Housed on Recycled Manure Solids Bedding: A Canadian Cohort Study
Source: Front Vet Sci. 2021 Sep 23;8:742868. doi: 10.3389/fvets.2021.742868 (PMC8495071; doi:10.3389/fvets.2021.742868)
Supplement: Supplementary file 2 [file Table_2.DOCX]

**Table S2.** Least square means clinical mastitis incidence estimates (in cases/100 cow-year) and incidence ratio (IR) between 26 RMS farms and 60 straw-bedded farms and computed using a binomial negative model and using and alternative computation of the animal-time at risk offset (i.e., using the number of days between first and last sample as time at risk). Models were adjusted for confounding by housing type, time since the last renovation of the stalls, bedding thickness, and herd size. Means within a row with different superscripts are statistically different.

| Clinical mastitis category | Estimated incidence  in cases/100 cow-year (95%CI) | | IR (95%CI) |
| --- | --- | --- | --- |
|  | RMS farms | Straw farms |  |
| All clinical mastitis | 26.5 (19.2, 36.6)^a^ | 46.2 (30.2, 70.8)^b^ | 0.6 (0.3, 1.0) |
| Severe clinical mastitis | 10.2 (6.9, 15.0) | 13.6 (7.1, 26.1) | 0.7 (0.3, 1.6) |
| By bacterial species involved |  |  |  |
| *Klebsiella pneumoniae* | 3.4 (1.6, 7.1)^a^ | 0.6 (0.2, 1.6)^b^ | 5.9 (1.6, 21.2) |
| *Streptococcus dysgalactiae* | 1.1 (0.5, 2.1) | 1.9 (0.8, 4.4) | 0.6 (0.2, 1.7) |
| *Escherichia coli* | 1.4 (0.7, 2.8) | 2.3 (1.1, 4.6) | 0.6 (0.3, 1.5) |
| *Streptococcus uberis* | 1.4 (0.5, 3.5) | 3.1 (1.3, 7.2) | 0.4 (0.1-1.5) |
| *Staphylococcus aureus* | 0.8 (0.3, 2.2) | 1.9 (0.7, 5.4) | 0.4 (0.2, 0.9) |
